# MR‐Linac guided adaptive stereotactic ablative body radiotherapy for recurrent cardiac sarcoma with mitral valve bioprosthesis – a case report

**DOI:** 10.1002/jmrs.669

**Published:** 2023-03-08

**Authors:** Vikneswary Batumalai, Madeline Carr, Michael Jameson, David Crawford, Urszula Jelen, Claire Pagulayan, Tania Twentyman, Angela Hong, Jeremy de Leon

**Affiliations:** ^1^ GenesisCare Sydney New South Wales Australia; ^2^ School of Clinical Medicine, Faculty of Medicine and Health UNSW Sydney Sydney New South Wales Australia; ^3^ Centre for Medical and Radiation Physics University of Wollongong Wollongong New South Wales Australia; ^4^ Faculty of Medicine and Health The University of Sydney Sydney New South Wales Australia

**Keywords:** adaptive radiotherapy, bioprosthesis, cardiac sarcoma, MR‐Linac guided radiation therapy, stereotactic ablative body radiation therapy

## Abstract

We present the first case in the literature of a 78‐year‐old woman with recurrent cardiac sarcoma adjacent to a bioprosthetic mitral valve treated with magnetic resonance linear accelerator (MR‐Linac) guided adaptive stereotactic ablative body radiotherapy (SABR). The patient was treated using a 1.5 T Unity MR‐Linac system (Elekta AB, Stockholm, Sweden). The mean gross tumour volume (GTV) size was 17.9 cm^3^ (range 16.6–18.9 cm^3^) based on daily contours and the mean dose received by the GTV was 41.4 Gy (range 40.9–41.6 Gy) in five fractions. All fractions were completed as planned and the patient tolerated the treatment well with no acute toxicity reported. Follow‐up appointments at 2 and 5 months after the last treatment showed stable disease and good symptomatic relief. Results of transthoracic echocardiogram after radiotherapy showed that the mitral valve prosthesis was normally seated with regular functionality. This study provides evidence that MR‐Linac guided adaptive SABR is a safe and viable option for the treatment of recurrent cardiac sarcoma with mitral valve bioprosthesis.

## Graphical abstract

We have successfully treated a unique case of undifferentiated cardiac sarcoma recurrence adjacent to a bioprosthetic mitral valve using MR‐Linac guided SABR. Overall, the patient had good symptomatic relief from MR‐guided adaptive radiotherapy with encouraging post‐treatment imaging results. The use of an MR‐Linac allowed the delivery of ultra‐hypofractionated (5 × 7 Gy) SABR safely and accurately and according to the patient's anatomy of the day.

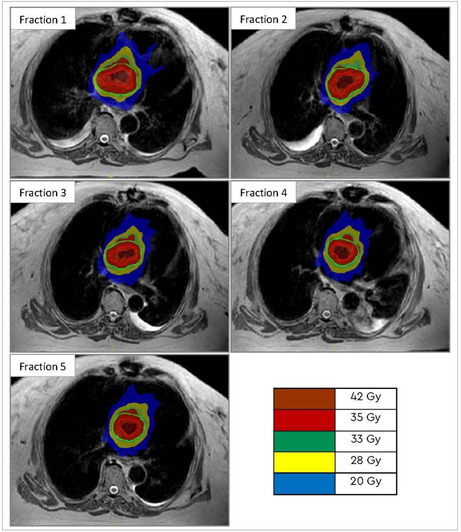

## Introduction

Primary cardiac sarcomas are rare in occurrence and the optimal treatment method is usually complete surgical resection.^1^ However, surgical resection often proves to be challenging or impossible due to the tumour's anatomic location.^2^ Other treatment options including chemotherapy and radiotherapy may be offered as part of multimodal treatment management to improve both progression‐free survival and overall survival.^3^ The current prognosis for patients with cardiac sarcoma is very poor, with a mean survival of 3 months to 1 year,^4^ and local recurrence and metastases occur frequently and often within 1 year.^5^

Over the past decade, stereotactic ablative body radiotherapy (SABR) with computerised tomography (CT)‐based image guidance on a standard linear accelerator (linac) has become an alternative treatment option for inoperable cardiac and pericardiac malignancies due to its capability to deliver highly ablative doses.^3^, ^6^, ^7^ However, there are challenges associated with delivering high‐dose radiotherapy to the heart including respiratory^8^ and cardiac motion^9^ and limiting dose to the heart. More recently, several studies have reported the safe use of magnetic resonance linear accelerator (MR‐Linac) guided SABR in the treatment of cardiac metastases^10^ and cardiac sarcomas.^11^, ^12^, ^13^ Advantages of the MR‐Linac system include better soft tissue contrast compared to CT‐based images, real‐time motion monitoring and the ability to conduct daily plan adaptation to deliver the most accurate plan of the day.

We report a unique case of an undifferentiated cardiac sarcoma recurrence adjacent to a bioprosthetic mitral valve treated with MR‐Linac guided adaptive SABR.

## Case Report

A 78‐year‐old woman presented with acute dyspnoea and intermittent palpitations in April 2020. She was found to be in acute heart failure with pulmonary oedema secondary to a large left atrial mass, with moderate to severe mitral inlet obstruction. The patient underwent a left atrial mass excision in May 2020. Intraoperatively, she was found to have a 4 cm mass occupying most of the left atrium. It was broad‐based and adherent to septum up to mitral annulus. This was excised and sent for pathological analysis. The patient received mitral valve replacement using a bioprosthesis (Perimount Aortic Magna Ease with Thermafix; Edwards Lifesciences, Irvine, CA, USA). The pathology results from the specimen excised revealed atrial spindle cell sarcoma (undifferentiated sarcoma).

The case was discussed in the sarcoma multi‐disciplinary team (MDT) meeting and was recommended for surveillance. Local recurrence in the left atrium was observed on a transthoracic echocardiogram 6 months after the surgery (November 2020). A mass was detected on the left atrium anterior wall measuring 33 × 31 mm in diameter and 36 mm in craniocaudal dimension, adjacent to the mitral valve prosthesis. A whole‐body positron emission tomography (PET) scan showed fluorodeoxyglucose (FDG) uptake of 6.1 in the lesion and no evidence of metastasis. The patient was referred to a radiation oncologist for treatment. The case was presented at an MR‐Linac peer‐review meeting, and the patient was recommended to receive MR‐Linac guided adaptive SABR with a dose of 35 Gy in five fractions, receiving treatment on non‐consecutive days.

Our investigation to safely treat the patient involved discussion with cardiologists, and it was noted that radiotherapy to the bioprosthetic mitral valve is not a concern as the valve does not contain any electronics unlike cardiac implantable electronic devices (CIED).

**Figure 1:**
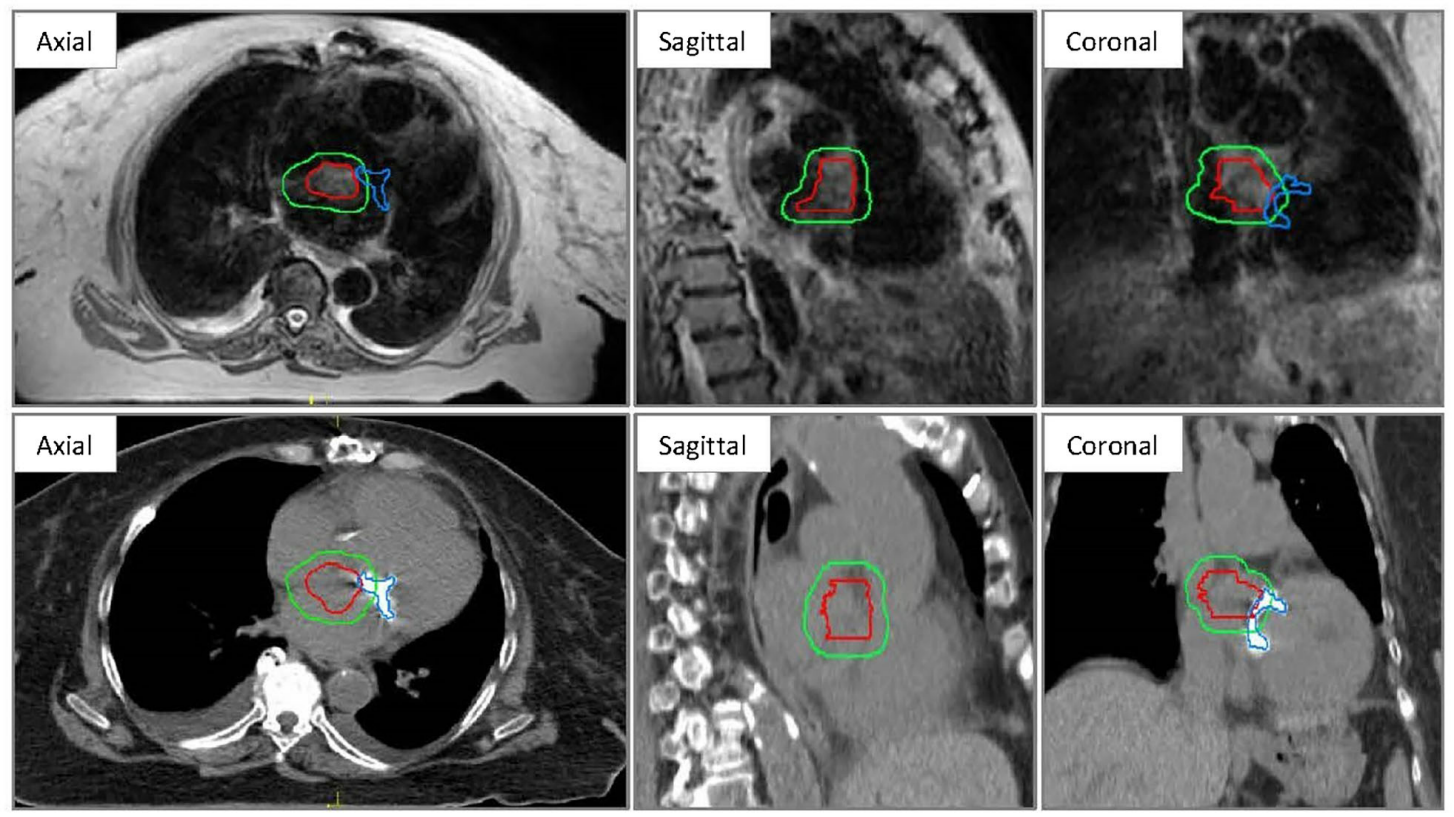
MR (top) and CT (bottom) reference scans showing gross tumour volume (red), planning target volume (green) and prosthetic valve (blue).

**Figure 2:**
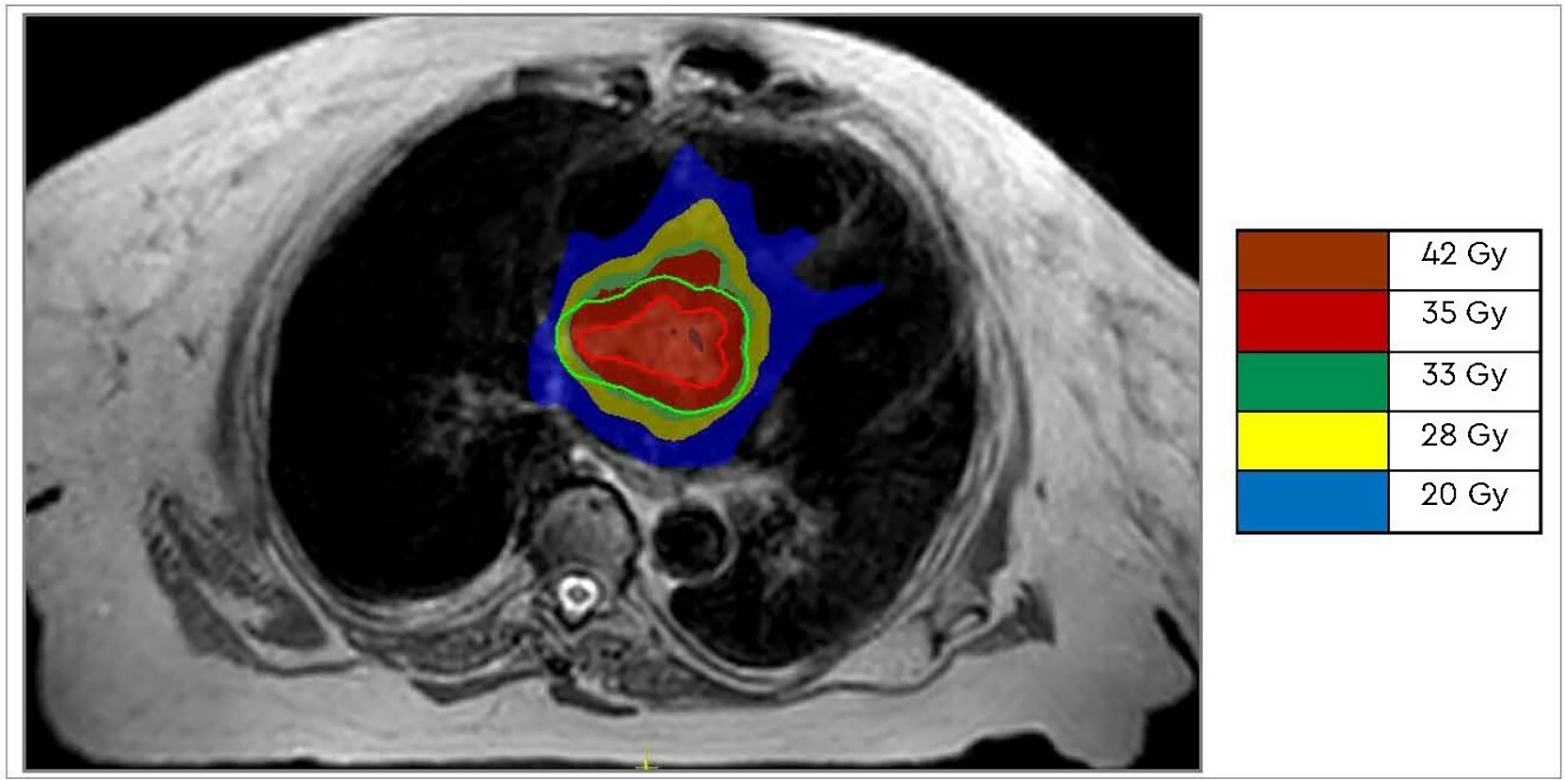
MR reference plan showing gross tumour volume (red), planning target volume (green) and isodose displayed in colour wash (20–42 Gy).

The patient received MR simulation on the 1.5 T Unity MR‐Linac (Elekta AB, Stockholm, Sweden) and a planning CT scan (Siemens Somatom Definition AS, Siemens Healthineers, Erlangen, Germany) acquired at 0.15 cm intervals without contrast. The patient was positioned supine with both arms elevated above the head and was supported by a wingboard. Abdominal compression was used to minimise the patient respiratory motion. A T2‐weighted MR scan was acquired on the MR‐Linac, and no visible artefacts were observed. There was a distinct difference in the size of delineated gross tumour volume (GTV) between the MR (18.66 cm^3^) and CT (15.03 cm^3^) scans (Fig. 1). As the MR images showed better soft tissue definitions of the target, delineation of the GTV was based on the visible lesion in the MR scan. Considering the expected respiratory and cardiac motion, an isotropic 7 mm margin around the GTV was chosen for the planning target volume (PTV). The heart, lungs, bronchi, spinal cord, aorta and oesophagus were contoured as organs at risk (OAR). The prosthetic valve was also contoured for visual representation and dose reporting. The plan was generated using the Monaco (version 5.40.01, Elekta AB) treatment planning system, with a 7MV flattening filter free, 9‐field step‐and‐shoot intensity‐modulated RT (IMRT) plan. The planning dose compliance was adapted from published guidelines^11^ and is summarised in Table 1. The reference plan (Fig. 2) resulted in 99 segments overall.

**Table 1:** Target and organs at risk constraints and mean dose achieved with range for five fractions.

Structure	Constraint	Dose achieved
		Mean (range)
GTV	D100% > 35 Gy	37.3 (37.1–37.7) Gy
PTV	D95% > 35 Gy	35.1 (35.0–35.1) Gy
Heart *excluding* PTV	D0.03cm^3^ < 38 Gy	36.9 (36.4–37.2) Gy
	Dmean <15 Gy	11.7 (10.0–13.9) Gy
Aorta	D0.03cm^3^ < 53 Gy	17.2 (16.3–19.1) Gy
	D10cm^3^ < 47 Gy	13.2 (12.6–13.7) Gy
Oesophagus	D0.03cm^3^ < 35 Gy	22.1 (20.7–24.1) Gy
	D5cm^3^ < 19.5 Gy	15.5 (14.6–16.0) Gy
Spinal cord	D0.03cm^3^ < 28 Gy	7.0 (6.2–7.8) Gy
	D0.35cm^3^ < 22 Gy	6.0 (4.6–6.8) Gy
Lungs	V13.5 < 37%	1.7 (1.0–2.6) %
	D1500cm^3^ < 12.5 Gy	0.8 (0.8–0.9) Gy
	Dmean <0.6 Gy	0.3 (0.3–0.4) Gy
Left bronchus	D0.03cm^3^ < 40 Gy	9.0 (4.7–13.6) Gy
	D5cm^3^ < 32 Gy	1.5 (1.4–1.7) Gy
Right bronchus	D0.03cm^3^ < 40 Gy	17.5 (14.5–19.6) Gy
	D5cm^3^ < 32 Gy	1.5 (1.3–1.6) Gy

**Figure 3:**
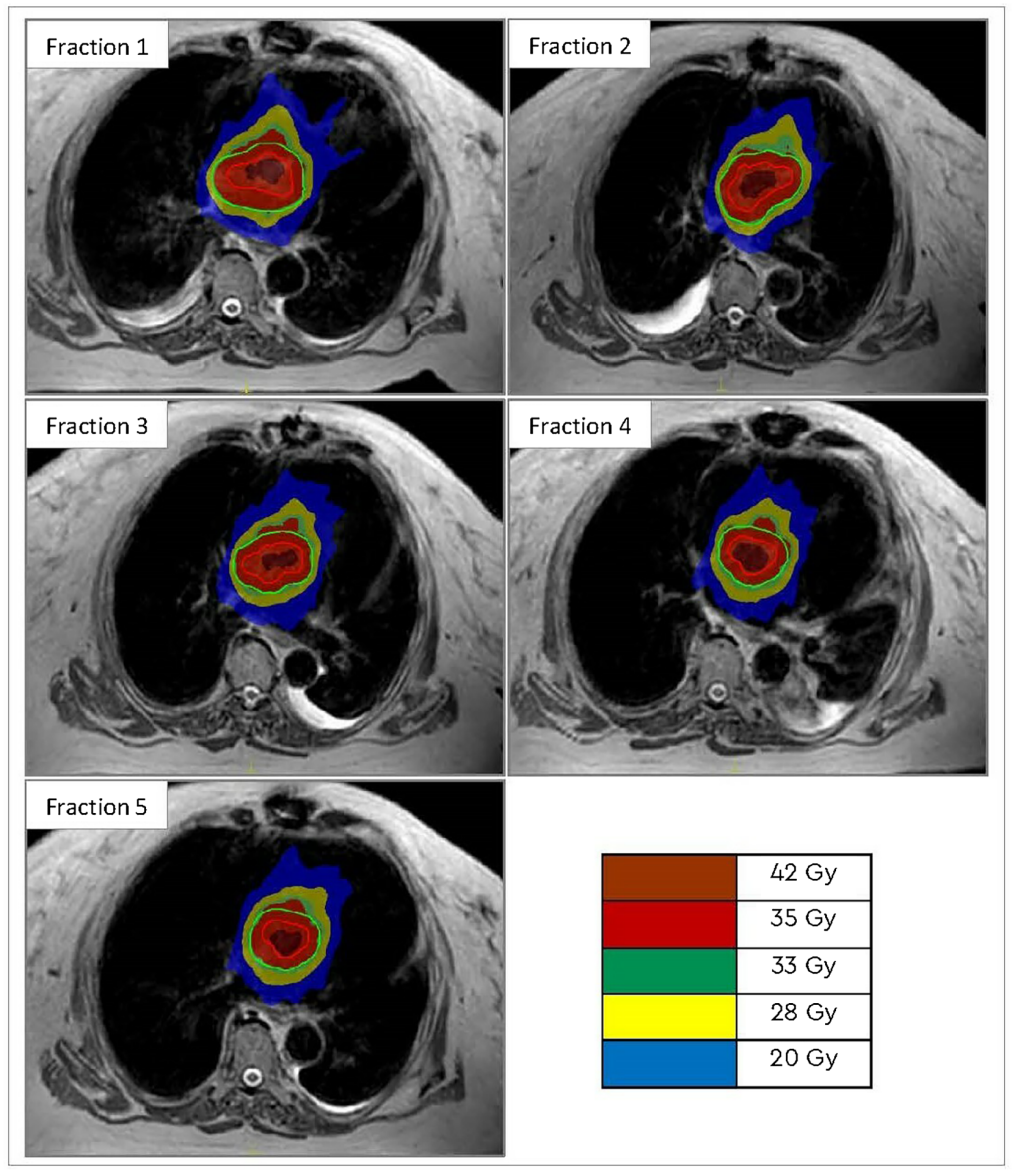
Daily MR‐guided adaptive radiotherapy showing gross tumour volume (red) and planning target volume (green) with the isodose displayed in colour wash (20–42 Gy).

**Figure 4:**
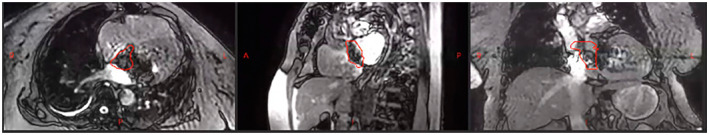
Example of cine MR images to estimate tumour motion, displayed using Elekta's pre‐clinical gating motion monitoring software. The gross tumour volume is displayed in red, whereas the 7 mm isotropic planning target volume expansion is shown in blue. Orthogonal directions are indicated on each of the images: anterior (A), posterior (P), left (L), right (R) and inferior (I).

An adapt‐to‐shape (ATS) strategy was employed for each fraction, where the daily MR scan is recontoured to adapt to the anatomy of the day.^14^ A T2‐weighted free‐breathing MR scan was acquired prior to each fraction and rigidly registered to the simulation MR. Deformable image registration was used to project the original set of contours onto the daily pre‐treatment MRI, and the PTV and OAR were re‐contoured by the radiation oncologist. Plan re‐optimisation was completed, and treatment was initiated after observing the patients motion using a balanced T1‐/T2‐weighted 2D cine MRI. Figure 3 shows the daily MR‐guided adaptive treatment for the patient. The GTV varied in size and shape between fractions due to deformation which occurred with the movement of the heart. However, monitoring the tumour motion prior to each treatment using cine MRI allowed for an assessment of expected tumour motion during treatment. Quantitative analysis of the free‐breathing GTV motion was conducted during MR simulation and fraction 1 using Elekta's pre‐clinical motion monitoring software.^15^ Figure 4 shows an example of cine MR images acquired pre‐treatment for motion monitoring.

Treatment was successfully completed with all the planned fractions treated on the MR‐Linac using the ATS protocol. The mean GTV size was 17.9 cm^3^ (range 16.6–18.9 cm^3^) and the mean PTV size was 66.9 cm^3^ (range 61.8–70.7 cm^3^). This resulted in a mean GTV dose of 41.4 Gy (range 40.9–41.6 Gy) and the mean PTV dose of 38.2 Gy (range 38–38.5 Gy) in five fractions. All target and OAR doses were met for all five fractions (Table 1). The prosthetic valve received a mean dose of 26.7 Gy (range 22.5–29.4 Gy). The average treatment time was 40 min (range 29–50 min). The GTV motion was found to stay within the bounds of the 7 mm isotropic margin of the PTV for pre‐treatment fraction 1 imaging, however, surpassed these bounds in the superior–inferior direction by a maximum of 1.21 mm at four different time points at MR simulation. This factor did not impact treatment decisions for this case, as the tumour was monitored in real time during treatment and would have been paused if found to be out of bounds.

**Figure 5:**
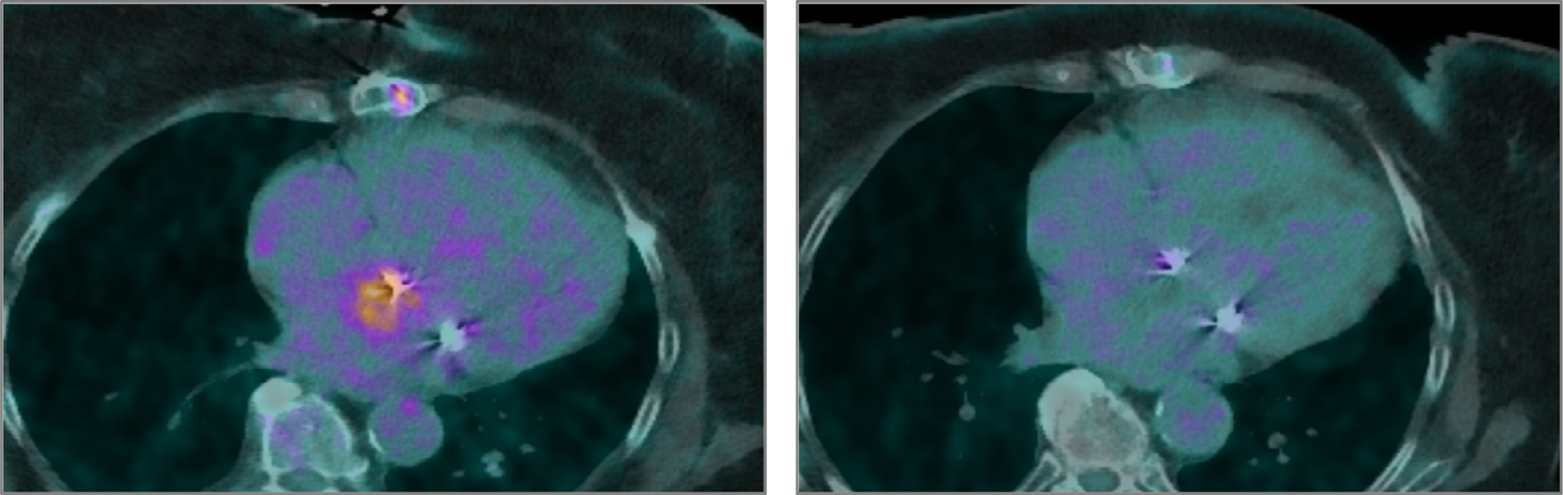
PET scan showing FDG standardised uptake value of 6.1 before treatment (left) and 4.2 two months after treatment (right) in the left atrial lesion.

The patient tolerated the treatment very well and reported no acute toxicity at the end of treatment. Four weeks after treatment, the patient reported no respiratory or cardiac symptoms, and no longer felt out of breath or had chest pain. A PET scan 2 months after treatment showed reduced FDG standardised uptake value (SUV) from 6.1 to 4.2 in the left atrial lesion (Fig. 5), with no evidence of disease elsewhere in the body. Transthoracic echocardiogram reported the mass to have reduced in diameter size to 28 × 15 mm. At 5‐month follow‐up after treatment, the patient still reported feeling asymptomatic and without any chest pain or shortness of breath. Echocardiogram showed that the mitral valve prosthesis remains normally seated with regular functionality, and the mass is stable in size. Overall, the patient had good symptomatic relief from MR‐guided adaptive radiotherapy with encouraging post‐treatment imaging results.

## Discussion

We have successfully treated a unique case of undifferentiated cardiac sarcoma recurrence adjacent to the bioprosthetic mitral valve using MR‐Linac guided SABR. The use of an MR‐Linac allowed the delivery of ultra‐hypofractionated (5 × 7 Gy) SABR safely and accurately. MR‐Linac guided adaptive SABR provided high‐quality images before and during treatment and allowed for a daily adaptive plan to account for the patient's anatomical changes. The average treatment on an MR‐Linac may be longer than on standard linac, however, the use of ultra‐hypofractionated SABR allowed delivery of the total dose in fewer fractions.

Several cases of MR‐Linac guided treatment for cardiac sarcomas have been reported in the literature.^11^, ^12^, ^13^ Corradini et al.^11^ reported on four patients treated using 0.35 T MR‐Linac (MRIdian, ViewRay Inc., Mountain View, CA, USA) with a mean GTV dose of 38.9 Gy (range 30.1–41.1 Gy) in five fractions. All treatments were completed as planned and patients tolerated the treatment well with reports of mild grade 1 or 2 symptoms (fatigue, dyspnea or mild chest pain) at early follow‐up.^11^ Pomp et al.^12^ reported the treatment of a patient with recurrent cardiac sarcoma after surgery treated using the Unity MR‐Linac with a prescription of 60 Gy in 12 fractions. A CT scan at 6 months after the last treatment showed stable disease.^12^ More recently, Noyan et al.^13^ reported the treatment of a patient using the Unity MR‐Linac in five fractions with a prescription dose of 25 Gy to the tumour bed and 30 Gy to the recurrent nodule. The patient tolerated the treatment well and had stable disease 2 months after treatment.^13^

Our case differs from those reported in the literature due to the presence of bioprosthetic mitral valve. Bioprosthetic heart valves were introduced for clinical use in the 1970s in order to overcome thromboembolic complications and the need for anticoagulation associated with mechanical prostheses.^16^ In preparation for the treatment, the bioprosthesis used for the mitral valve replacement was investigated for radiotherapy and MR safety. Notably, there has never been an adverse event reported in association with performing an MR scan in patients with bioprosthesis in the valve.^17^ It was observed from the product sheet from Edwards Lifesciences that artefacts can be seen up to 36 mm from the valve for GE Healthcare (Milwaukee, WI, USA) MR scanner sequences and 11.5 mm for Siemens Healthineers (Erlangen, Germany) MR scanner sequences on 3 T systems. From our experience, T2‐weighted MR sequence resulted in no visible artefacts. This allowed for better visualisation of the target and the surrounding tissues.

While the effects of radiation on CIED such as pacemaker and implantable cardioverter defibrillator is known,^18^ no data exist on the effect of radiotherapy on bioprosthetic heart valves. Discussion in the MDT meeting and cardiologists resulted in an understanding that radiotherapy to the bioprosthetic mitral valve is not a concern as the valve does not contain any electronics. Therefore, no additional dose constraint was applied to the bioprosthetic valve during the optimisation process. The valve was contoured for dose‐reporting purposes. Our finding showed that a mean dose of 26.7 Gy to the bioprosthetic mitral valve was safe and results of transthoracic echocardiogram at 2‐ and 5‐month post‐radiotherapy showed that the mitral valve prosthesis was normally seated with regular function.

## Conclusion

We presented the first case of recurrent cardiac sarcoma with mitral valve prosthesis treated with MR‐Linac guided SABR. The treatment was well tolerated by the patient with good symptomatic relief. With promising follow‐up results from our case study and those in the literature, MR‐Linac guided adaptive SABR should be considered for future inoperable primary or recurrent cardiac sarcomas.

## Conflict of Interest

The authors declare no conflict of interest.

## Consent

Patient consent for publication was obtained as part of the ADAPT‐MRL registry (ACTRN12621000385842).
